# Measuring Goal-Concordant Care Using Electronic Clinical Notes

**DOI:** 10.1001/jamanetworkopen.2025.18967

**Published:** 2025-07-03

**Authors:** Catherine L. Auriemma, Anne Song, Lake Walsh, Jason Han, Sophia Yapalater, Alexander Bain, Lindsay Haines, Stefania Scott, Casey Whitman, Stephanie Parks Taylor, Gary E. Weissman, Matthew J. Gonzales, Roshanthi Weerasinghe, Staci J. Wendt, Katherine R. Courtright

**Affiliations:** 1 Division of Pulmonary, Allergy, and Critical Care, Hospital of the University of Pennsylvania, Philadelphia; 2 Palliative and Advanced Illness Research Center, University of Pennsylvania, Philadelphia; 3 Department of Medicine, University of Pennsylvania, Philadelphia; 4 Leonard Davis Institute of Health Economics, University of Pennsylvania, Philadelphia; 5 Division of Internal Medicine and Primary Care, Brigham and Women’s Hospital, Boston, Massachusetts; 6 Department of Surgery, University of Pennsylvania, Philadelphia; 7 Supportive Oncology and Palliative Care Program, Fox Chase Cancer Center, Temple University Health System, Philadelphia, Pennsylvania; 8 Division of Pulmonary and Critical Care, New York University-Langone, New York, New York; 9 Division of Hospital Medicine, University of Michigan, Ann Arbor; 10 The Institute for Human Caring at Providence, Gardena, California; 11 Providence Health Research Accelerator, Portland, Oregon

## Abstract

**Importance:**

Goal-concordant care (GCC) is recognized as the highest quality of care and most important outcome measure for serious illness research, yet practical methods for measuring it are lacking.

**Objective:**

To measure GCC using clinical notes in patients’ medical records.

**Design, Setting, and Participants:**

This longitudinal cohort study involved a retrospective medical record review in 3 urban hospitals in a single health system. Participants included adults with a hospital encounter of 3 or more days between April 1 and July 31, 2019, and 50% or higher predicted 6-month mortality risk. Data abstraction occurred from July 2021 through June 2022.

**Exposure:**

Acute care hospitalization and a 50% or higher predicted 6-month mortality risk.

**Main Outcomes and Measures:**

Pairs of clinicians independently reviewed clinical notes from admission through 6 months or death to classify the care received during each epoch between patients’ documented goals of care (GOC) discussions, into 1 of 4 categories: (1) comfort focused, (2) maintain or improve function, (3) life extension, or (4) unclear. The GOC discussions had been previously classified using the same 4 categories. The primary study outcome was GCC, defined as the alignment of classification of care received and GOC. Secondary outcomes included goal-discordant care, if GOC and care-received classifications were misaligned, and uncertain concordance, if either care received or GOC was classified as unclear or GOC were not documented. Interrater reliability for classification of care received was assessed using Cohen κ statistics.

**Results:**

Among 109 patients (53 female [49%]), the median (IQR) age was 70 (63-79) years. The most common serious illnesses were cardiac disease (76 patients [70%]), metastatic cancer (50 patients [45%]), and chronic kidney disease (42 patients [39%]). Interrater reliability for care-received classification was almost perfect (95% interrater agreement, Cohen κ = 0.92; 95% CI, 0.86-0.99). A total of 398 epochs of care were identified, 198 (50%) of which were classified as goal concordant. Of the remaining 200 epochs, 74 (19%) were classified as goal discordant and 126 (32%) of uncertain concordance. During at least 1 epoch of care over the 6-month follow-up, 85 patients (78%) received care of uncertain concordance and 43 (39%) received goal-discordant care.

**Conclusions and Relevance:**

In this cohort study of seriously ill adults, GCC was measured using clinical notes alone. These findings can inform automated text-based classification methods to improve the efficiency and scalability of this method and facilitate pragmatic and reliable measurement of GCC in serious illness research and quality improvement efforts.

## Key Points

**Question:**

Can clinical notes in the electronic health record be used to reliably measure goal-concordant care?

**Findings:**

In this longitudinal cohort study among 109 patients with serious illness and limited prognoses, clinicians reviewed and classified 398 epochs of care as goal concordant (50%), goal discordant (19%), or of uncertain concordance (32%) with nearly perfect interrater agreement for categorizing the type of care received (Cohen κ = 0.92).

**Meaning:**

These findings suggest that using electronic clinical notes to measure goal-concordant care is feasible, laying the groundwork for future automated text-based classification methods to improve reliability and pragmatism of measuring goal-concordant care for clinical and research use at scale.

## Teaser

This cohort study uses clinical notes in electronic health records to measure goal-concordant care for patients with serious illness and high predicted 6-month mortality.

## Introduction

Goal-concordant care (GCC) is medical care that aligns with and promotes patients’ goals of care (GOC), including preferences regarding treatment intensity, functional outcomes, and longevity. GCC is widely considered the highest quality of care^1,2^ and is recognized as a key priority of numerous medical specialties and societies.^3,4,5,6^ GCC has also been identified as the most important outcome for assessments of serious illness care, advance care planning, and palliative and end-of-life care intervention studies.^7^ However, there is no consensus or validated method for how to measure GCC,^8,9,10,11^ impeding efforts to evaluate the effectiveness of serious illness quality improvement and research interventions.

Prior approaches to measuring GCC have focused on proxy assessments, such as communication timing or quality,^9^ or narrowly defined populations, such as patients with advanced cancer.^8,12,13^ Although assessments reported by patients, caregivers, or clinicians^14,15^ are important perspectives on GCC delivery to consider, they often are limited by desirability, recall, and selection biases. Furthermore, collection of these assessments from patients with serious illness or their caregivers is often infeasible at scale, leading to substantial data missingness. A framework for measuring GCC using clinical documentation in the electronic health record (EHR)^10^ has the advantages of allowing for longitudinal assessment, using a data source that contains a wealth of information on care delivery, and is where GOC discussions during routine care are most often documented.^1^

Knowledge of patients’ GOC is necessary when trying to determine whether GCC has been delivered, although it is insufficient for this task without also knowing the type of care that they received. The need for both parts of the GCC equation contributes, in part, to the challenges of measuring it from EHR data.^12,16,17^ Thus, building upon our prior work to operationalize an adapted framework^10,18^ using data from clinical notes to classify GOC, we conducted a proof-of-principle, longitudinal medical record review study to classify the type of care received with the primary aim of measuring GCC among patients with limited prognoses. We secondarily explored patient characteristics associated with receipt of GCC.

## Methods

### Study Design and Setting

We conducted a longitudinal, retrospective medical record review cohort study among seriously ill patients admitted to 1 of 3 urban, academic-affiliated hospitals in the University of Pennsylvania Health System (UPHS) between April 1, 2019, and July 31, 2019. Data abstraction occurred from July 2021 through June 2022. The UPHS institutional review board considered this study exempt from review and informed consent because the data were deidentified, in accordance with 45 CFR §46.104, category 4. This study follows Strengthening the Reporting of Observational Studies in Epidemiology (STROBE) reporting guidelines for cohort studies.^19^

### Study Population

**Figure 1.**
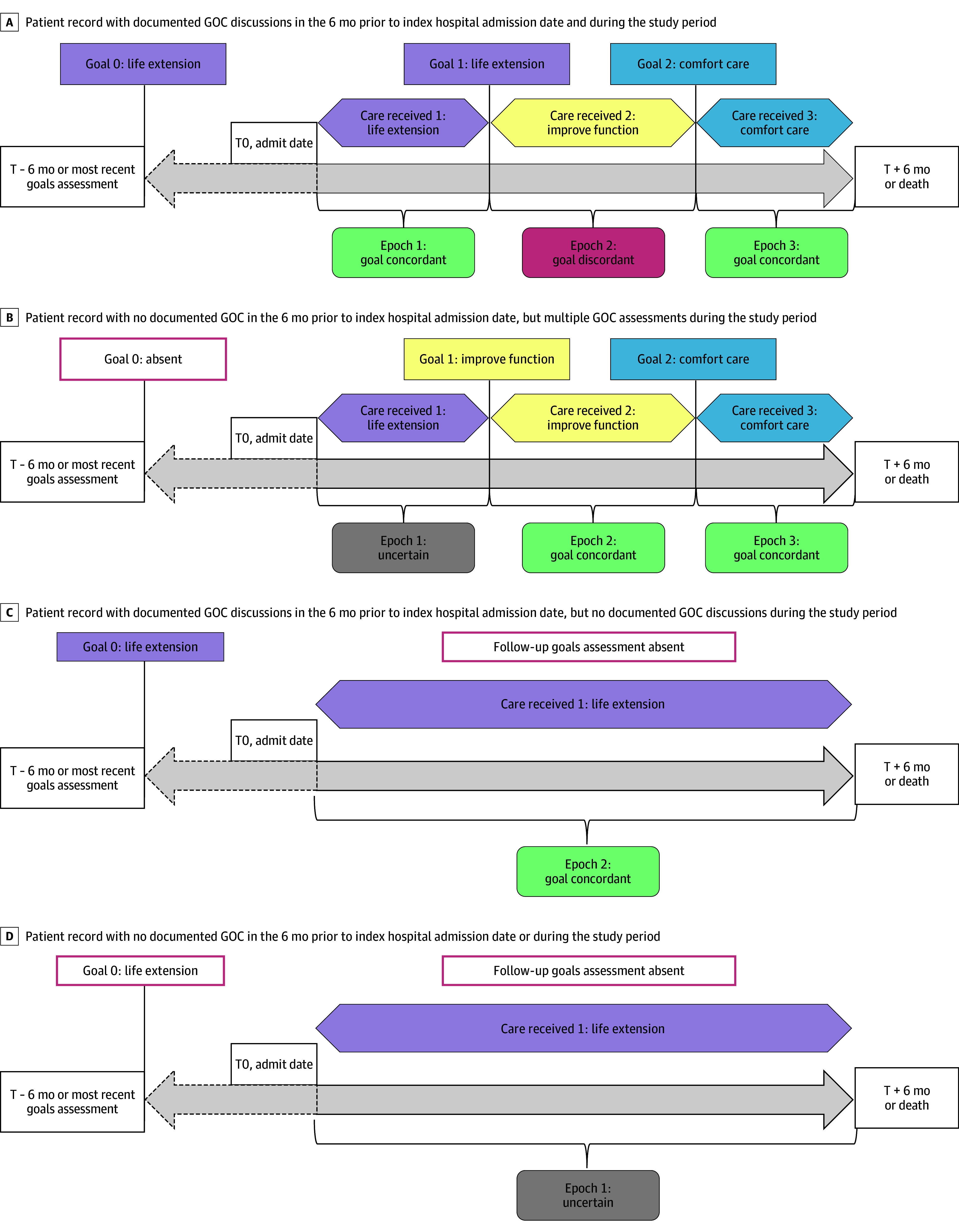
Assessments of Goals of Care (GOC) and Care Received Over Time, Illustrating Delineation of Epochs and Identification of Goal-Concordant, Goal-Discordant, and Uncertain Care Each panel illustrates an example of a patient.

Study population details were published previously.^18^ Briefly, we identified adults aged 18 years or older with 50% or higher predicted risk of 6-month mortality at admission,^20^ a length of stay of 3 or more calendar days, and 1 or more prior encounters in the health system within the preceding 12 months. We used a random sampling approach, stratified evenly across high (50%-74%) vs very high (≥75%) 6-month mortality risk to identify 120 patient records. One duplicate patient record was identified and removed. Ten records were randomly selected for use in piloting, with the remainder serving as the final analytic sample. Patients’ self-reported sex, race, and ethnicity data were obtained from the UPHS clinical data warehouse and are included here to enable assessment of representativeness of the patient cohort. As described previously^18^ and detailed in Figure 1, all documented GOC discussions^21^ found in inpatient, ambulatory, and home-care encounter notes for the cohort from the index admission through 6 months or death had been previously used to classify patients’ goals into 1 of 4 categories: (1) comfort focused, (2) maintain or improve function, (3) life extension, or (4) unclear. The most recent documented GOC discussion within the 6 months prior to admission was used to establish baseline GOC at admission, and if no such prehospital GOC discussion existed, then baseline goals were categorized as unclear.

### Data Collection and Variable Definitions

#### Patient Characteristics and Clinical Data

Patients’ sociodemographic data were obtained from the UPHS EHR (Epic) data warehouse: self-reported binary sex, race, ethnicity, religion, and primary language. Palliative care consultations were identified by the presence of a signed order during the index hospital encounter. We also collected readmissions to any UPHS hospital, home care visits, and referrals to home or outpatient palliative care during the 6-month follow-up.

#### Care Received Classification

We assessed care received longitudinally from the date of hospital admission through death or 6 months, whichever occurred first. The 6-month follow-up period was divided into epochs, with time 0 of the first epoch being admission date for all patients and the beginning of the next epoch defined by the date of subsequently documented GOC discussions (Figure 1). If a patient had no GOC discussion documented during the study period, their care was assessed as a single epoch from admission through end of follow-up. This design resulted in a different number of epochs for individual patients.

Each epoch was reviewed by a pair of clinician reviewers blinded to GOC categorizations to classify patients’ care received during that time. Reviewers included a medical intensivist (C.L.A.), a critical care hospitalist (A.B.), a hospice and palliative medicine fellow (A.S.), and a fourth-year medical student and internal medicine resident (L.W.), all of whom trained together on a set of 10 records to promote consistency in applying the classification framework. All advance care planning notes, interdisciplinary palliative care consultant notes, and other practitioner notes in which a GOC discussion had been identified were excluded from the care-received assessment to avoid influencing reviewers’ classifications.

Reviewers were instructed to determine the perceived intention of the medical treatments delivered during each GOC epoch to classify the care received using the same 4 categories as GOC (Table 1). To aid in this process, reviewers used a checklist of common procedures and treatments to identify within each epoch. Reviewers were also encouraged to describe in open-ended form any additional care noted during the epoch, as well as reasoning for their ultimate classification. *Life extension* included therapies delivered with the primary goal to prolong life, regardless of whether treatments might exacerbate pain, discomfort, or dysfunction in the short or long term. *Maintain or improve function* included therapies delivered with the goal of promoting the patient’s physical or cognitive function, even if treatments might exacerbate pain, discomfort, or dysfunction in the short term. *Comfort-focused care* included therapies with the primary goal of relieving or avoiding suffering, including withholding or withdrawing life-prolonging therapies.

**Table 1.** Care-Received Classification, Definitions, and Example Descriptions From Data Abstraction, Including Excerpts From the Electronic Health Record

Category and definition	Example descriptions from data abstractors
Life extension: care plan focuses on maximizing the patient’s longevity without limitations on care. Extending longevity or survival are prioritized over maximizing function or comfort.	“Direct admission for kidney transplant (under general anesthesia, intubation, central line, arterial line), also underwent peritoneal dialysis catheter removal intra-operatively. Extubated post-operatively prior to transfer to floor, eventually discharged home.”
	“Metastatic breast cancer complicated by malignant effusions, ventilator-dependent respiratory failure secondary to pneumonia. Underwent tracheostomy and feeding tube placement. Continued on vasopressors, total enteral nutrition, and ventilator wean. Readmitted for multifactorial respiratory failure to acute care hospital, then transferred to long-term acute care hospital.”
	“Decompensated cirrhosis admitted with AKI. Previously on transplant list, deactivated on admission temporarily given functional decline. Nasogastric tube placed for supplemental enteral nutrition. Developed worsening labs concerning for infection, started antibiotics. Underwent diagnostic/therapeutic paracentesis and thoracentesis. Admitted to medical ICU for worsening respiratory failure on noninvasive ventilation, then intubated. Goal of stabilization in order to consider living donor liver transplant.”
Maintain or improve function: care plan focuses on maintaining or improving cognitive or physical function by preventing or reversing dysfunction, even if that medical care would increase discomfort; however, care that would increase survival or longevity without preservation or improvement in function is generally avoided.^a^	“Admitted to neuro ICU for acute ischemic stroke, underwent carotid endarterectomy; transferred post-op to ICU for frequent neuro checks; arterial line continued. Continued chronic intermittent hemodialysis. Discharged to acute rehab; readmitted due to anemia concerning for gastrointestinal bleed complicated by non-ST elevation myocardial infarction. Patient stated DNR/DNI. Underwent esophagogastroduodenosocopy (under monitored anesthesia care), which found 2 arterio-venous malformations, treated with argon plasma coagulation; discharged again to acute rehab.”
	“Admitted to acute rehab. Patient reported to provider she would like time-limited trial of intubation, so code status changed to full code. Improved activities of daily living with physical, occupational, and speech therapy and discharged home. Underwent outpatient colonoscopy (under monitored anesthesia care). Underwent outpatient interventional radiology arterio-venous fistulogram; rapid response called for hypertension, chest pain, nausea, and transferred to ED, then discharged home.”
	“Patient continued on ventilator via tracheostomy. Goal of treating pain and anxiety without invasive procedures. Code status switched [from full code] to DNR, may intubate. Not yet ready for palliative ventilator liberation, wants to go to vent-capable facility and maximize quality of life. Readmitted to medical ICU with respiratory failure, started on antibiotics. Air hunger was treated with morphine and remained on mechanical ventilation throughout admission. Discharged to skilled nursing facility.”
Comfort-focused: care plan focuses on maximizing comfort and relieving or avoiding suffering. Includes seeking interventions to promote comfort and avoiding interventions that would increase discomfort, even at the expense of decreasing longevity.^a^	“PICC line placed. Code status changed to DNR/DNI. Tachyarrhythmia treatments disabled from pacemaker and implanted defibrillator. Continued on palliative milrinone. Discharged to home hospice.”
	“Code status changed to ‘DNR, may intubate.’ Plan for home hospice. Only receiving steroids, pain meds, and bowel regimen. Keeping foley for comfort despite risk of infection.”
	“Transferred to inpatient hospice. ‘Suspected wall port may be seeded with bacteria as patient continued to experience rigors and expiratory wheezes when IV medications were administered through port. Given that patient had transitioned to comfort focused care, port removal was deferred as it’s an invasive procedure.’”

Abbreviations: AKI, acute kidney injury; DNI, do not intubate; DNR, do not resuscitate; ED, emergency department; ICU, intensive care unit; IV, intravenous; PICC, peripherally inserted central catheter.

^a^ If reviewers were having difficulty distinguishing between life extension and maintain or improve function for a particular epoch, they were advised to use code status to distinguish between the 2 categories as follows: no limitations on resuscitation was considered more consistent with life extension, whereas documented plans to limit resuscitation (DNR and/or DNI) were considered more consistent with maintain or improve function.

Reviewers were instructed to assign a single best-fit category for the overall care received during each epoch. Specific considerations were given to time-limited trials of critical care or other invasive interventions. For example, if the perceived intent was to identify and treat reversible issues to regain some baseline function, this would be considered consistent with maintain or improve function rather than life extension. Similarly, code status was intentionally not considered a primary defining feature of any category. However, reviewers could use code status to help distinguish between life extension (ie, full code) and maintain or improve function (ie, do-not-attempt resuscitation or do-not-intubate ) when the intent of care delivered was otherwise unclear.

Reviewers reported their confidence in the care-received assessment for each epoch using a 5-point Likert scale (1 = not confident at all, 3 = moderately confident, and 5 = very confident). Reviewer pairs were blinded to each other’s categorizations until both had independently completed their care-received assessments. Interrater reliability was calculated after independent coding. Next, disagreements were discussed and resolved within reviewer pairs. If consensus could not be reached, physicians with expertise in critical care and palliative care (C.L.A, when not part of the reviewer pair, or K.R.C.) performed an independent review and adjudication.

#### GCC Outcome

The primary outcome was GCC. For each epoch, care was defined as goal-concordant if there was agreement between GOC and care-received assessments (eg, both GOC and care received classified as life extension), and neither was classified as unclear. Goal-discordant was defined as misalignment between GOC and care-received assessments (eg, GOC categorized as life extension, and care received categorized as comfort focused), and neither was classified as unclear. Uncertain concordance was defined when there was no GOC discussion, or when either GOC or care received was classified as unclear. Finally, we also assessed GCC as a binary outcome, high rate of GCC, defined at the patient level as having 75% or more of epochs classified as goal concordant.

### Statistical Analysis

We calculated the raw agreement percentage between reviewers for classification of care received and measured interrater reliability using the Cohen κ statistic. The primary unit of analysis for GCC was an epoch. For individual patients, we also calculated the proportion of epochs classified as receiving GCC, goal-discordant care, and uncertain concordance of care. We used bivariable logistic regression, χ^2^ tests, and Fisher exact tests to identify associations between prespecified patient baseline characteristics (age, sex, race, religion, insurance status, chronic comorbidities, index hospital-stay risk of 6-month mortality, admitting service, and palliative care referral) and a high rate of GCC, GOC classification, and any receipt of goal-discordant care. We also assessed the association between care-received classification and reviewers’ confidence rating (1-2 [low] vs 3-5 [moderate-to-high]) and between GOC classification and receipt of GCC using χ^2^ tests. Statistical significance was set at an α = .05 for 2-tailed tests. Statistical analyses were conducted using Stata statistical software version 17 (StataCorp).

## Results

### Patient Cohort and Data Missingness

The cohort consisted of 109 patients (median [IQR] age, 70 [63-79] years; 53 female [49%]). The most common serious illness comorbidities were cardiac disease (76 patients [70%]), metastatic cancer (50 patients [45%]), and chronic kidney disease (42 patients [39%]). Additional demographic and clinical characteristics, as well as the classification of GOC discussions, were reported previously^18^ and are described in eTable 1 in Supplement 1. Seven individuals (6%) survived the index hospital stay and were lost to follow-up at UPHS during the study period.

### Documented GOC Discussions

**Figure 2.**
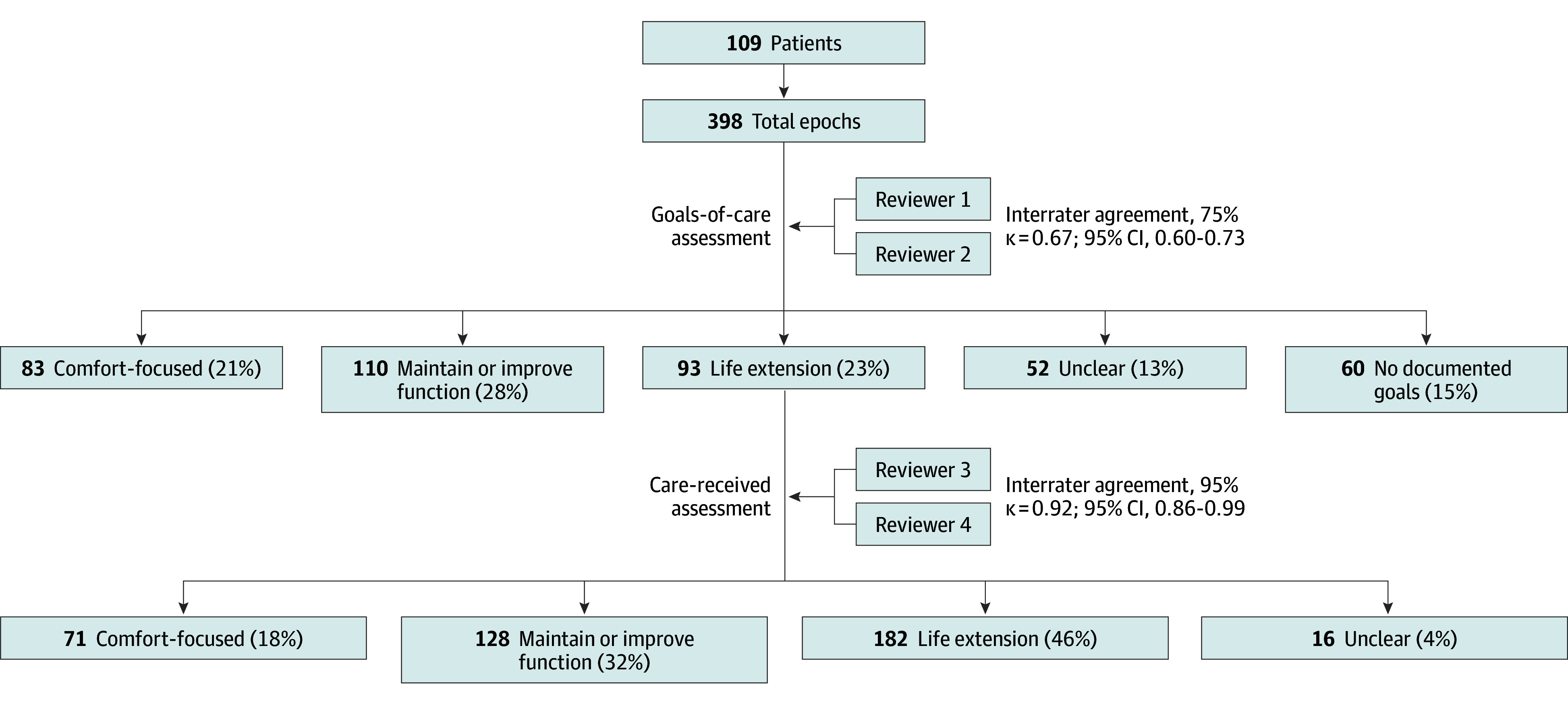
Study Flow and Classification of Goals-of-Care and Care Received

Forty-nine patients (45%) had a baseline GOC discussion documented in the 6 months prior to the index admission. Overall, there were 398 epochs derived from a total of 289 GOC discussions among 83 patients (76%); the remaining 26 patients (24%) had no GOC discussions documented during the study period (Figure 2). Compared with patients without a documented GOC discussion, those with at least 1 documented GOC discussion more often had very high (≥75%) vs high (50%-74%) predicted risk of 6-month mortality (47 patients [57%] vs 8 patients [31%]; *P* = .02; χ^2^ = 5.30) and a palliative care referral during the admission (43 patients [52%] vs 0 patients [0%]; *P* < .001, Fisher exact test). GOC discussions were also more commonly documented among patients admitted to a medical vs surgical service (74 patients [82%] vs 9 patients [47%]; *P* = .001; χ^2^ = 10.49). Fifty patients (45.9%) died during the study period, 49 of whom (98%) had 1 or more GOC discussion.

### Categorization of Care Received

**Figure 3.**
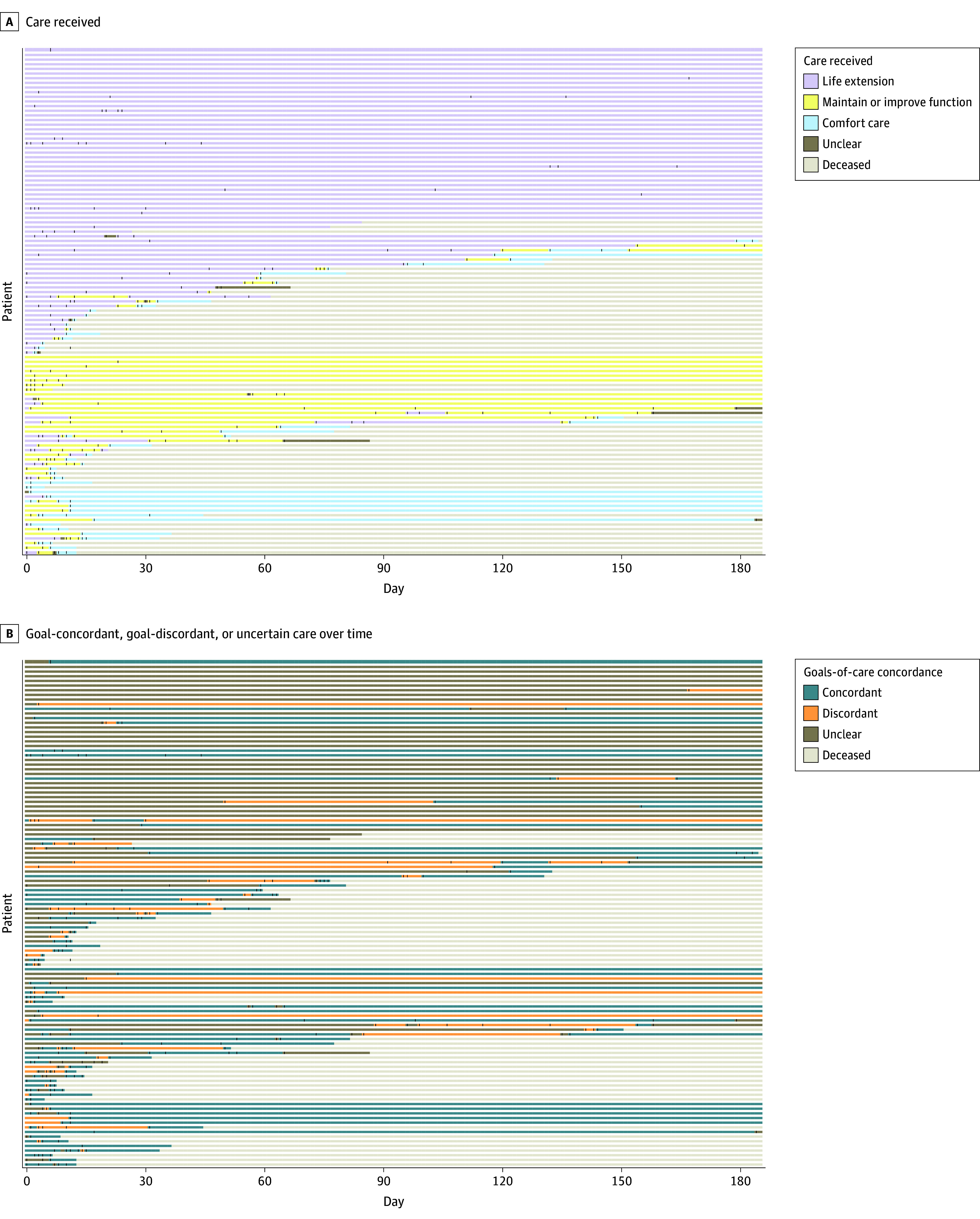
Tile Plots Showing Frequency and Classification of Patients’ Care Received and Goal-Concordant, Goal-Discordant, or Uncertain Care Over Time Plots were made with Ggplot in R software version 4.3.0 (R Project for Statistical Computing). Each patient is represented on the y-axis. Dashes denote individual goals-of-care discussions for each patient during the study period.

Interrater reliability for classifying care received was nearly perfect (95% interrater agreement, Cohen κ, 0.92; 95% CI, 0.86-0.99). Overall, the most common category of care received was life extension (183 epochs [46%]), followed by maintain or improve function (128 epochs [32%]), comfort focused (71 epochs [18%]), and unclear (16 epochs [4%]) (Figure 3). The median (IQR) reviewer confidence score for care-received classifications was 4 (3-5), and interrater reliability remained high within the low confidence (κ, 0.86; 95% CI, 0.71-1.00) and moderate-to-high confidence (κ, 0.92; 95% CI, 0.86-0.99) subgroups. Reviewers were more often moderately-to-very confident when categorizing care received as comfort focused compared with life extension (132 epochs [93%] vs 312 epochs [85%]; *P* = .02; χ^2^ = 5.53) or maintain or improve function (132 epochs [93%] vs 210 epochs [82%]; *P* = .003; χ^2^ = 9.02). Table 1 includes representative excerpts of clinical text for each care-received category. Frequency of specific treatments or procedures that patients received are shown in Table 2, with the most common being an invasive diagnostic or minor therapeutic procedure (80 patients [73%]) and physical or occupational therapy (74 patients [68%]).

**Table 2.** Specific Diagnostic and Therapeutic Interventions Assessed During Record Review and Frequency of Occurrence

Intervention	Interventions, No. (%)				
	Across 109 patients	Across 398 epochs	Across 183 life-extension epochs	Across 128 maintain or improve function epochs	Across 71 comfort-focused epochs
Invasive diagnostic or minor therapeutic procedure^a^	80 (73)	144 (36)	99 (54)	41 (32)	4 (6)
Physical and/or occupational therapy (inpatient or outpatient)	74 (68)	115 (29)	75 (41)	40 (31)	0 (0)
Do not resuscitate or do not intubate order	54 (50)	65 (16)	6 (3)	37 (29)	22 (31)
Admission to intensive care unit	43 (39)	54 (14)	40 (22)	14 (11)	0 (0)
Hospice enrollment	38 (35)	51 (13)	0 (0)	4 (3)	47 (66)
Chemotherapy	31 (28)	48 (12)	41 (22)	7 (5)	0 (0)
Invasive palliative procedure^b^	26 (24)	43 (11)	20 (11)	15 (12)	8 (11)
Intubation with mechanical ventilation	19 (17)	38 (10)	29 (16)	9 (7)	0 (0)
Comfort-care only order	31 (28)	35 (9)	0 (0)	0 (0)	35 (49)
Admission to short-term skilled nursing facility or acute rehabilitation center	24 (22)	30 (8)	21 (11)	9 (7)	0 (0)
Dialysis	13 (12)	25 (6)	18 (10)	6 (5)	1 (1)
Home palliative care consult	16 (15)	24 (6)	11 (6)	13 (10)	0 (0)
Surgical procedure	22 (20)	22 (6)	19 (10)	3 (2)	0 (0)
Palliative extubation and/or ventilator withdrawal	6 (6)	6 (2)	0 (0)	1 (1)	5 (7)
Advanced cardiac life support or cardiopulmonary resuscitation	6 (6)	6 (2)	4 (2)	2 (2)	0 (0)
Deactivation of internal cardiac defibrillator or pacemaker	1 (1)	1 (0.3)	0 (0)	0 (0)	1 (1)

^a^ Invasive diagnostic or minor therapeutic procedures included central venous catheter, feeding tube, thoracentesis, paracentesis, lumbar puncture, extracorporeal membrane oxygenation, chest tube insertion, biopsy, bronchoscopy, and interventional radiology procedure.

^b^ Invasive palliative procedures included thoracentesis, paracentesis, pleural catheter placement, nerve block, venting gastrostomy tube placement, and lumbar puncture to relieve elevated intracranial pressure.

### GCC, Goal-Discordant Care, and Uncertain Care

Of the 398 epochs, GCC was identified in 198 (50%), and goal-discordant care was identified in 74 (19%) (Figure 3; eTable 2 in Supplement 1). Receipt of goal-discordant care was most common when the documented goals were categorized as maintain or improve function compared with life-extension (38 epochs [35%] vs 18 epochs [19%]; *P* = .02; χ^2^ = 5.82) or comfort-focused goals (38 epochs [35%] vs 18 epochs [22%]; *P* = .05; χ^2^ = 3.80). Uncertain concordance was identified in 126 epochs (32%) and was due to either the lack of a preadmission baseline GOC discussion (60 epochs [48%]), a GOC discussion being classified as unclear (52 epochs [41%]), and/or care received being classified as unclear (16 epochs [13%]).

### Patient-Level Analyses

Overall, patients had a median (IQR) of 3 (2-5) epochs in the 6-month follow-up period. The median (IQR) duration of each epoch between GOC discussions was 7 (2-115) days. Patients received GCC in a median (IQR) of 40% (0%-75%) of epochs. Eighty patients (73%) received GCC during at least 1 epoch. Patients who received an inpatient palliative care consultation were more likely to receive a high rate (≥75% of epochs) of GCC compared with those who did not (19 patients [44%] vs 9 patients [14%]; *P* < .001; χ^2^ = 12.73). Patients with metastatic cancer were also more likely to receive a high rate (75% of epochs) of GCC compared with those without metastatic cancer (19 patients [38%] vs 9 patients [15%]; *P* = .007; χ^2^ = 7.34). Patients with metastatic cancer also more commonly received inpatient palliative care consultation than those without (28 patients [56%] vs 15 patients [25%]; *P* = .001; χ^2^ = 10.59). Only 8 patients (7%) received GCC during every epoch; no patient characteristics were associated with receiving 100% GCC. Among the 50 patients (46%) who died, 42 (84%) were receiving GCC at the time of death, 5 (10%) had care of uncertain concordance, and 3 (6%) goal-discordant care.

Forty-three patients (39%) received goal-discordant care during 1 or more epochs, and this was associated with being admitted to a medical compared with a surgical service (40 patients [44%] vs 3 patients [16%]; *P* = .02; Fisher exact test) and having a diagnosis of metastatic cancer (25 patients [50%] vs 18 patients [31%]; *P* = .04; χ^2^ = 4.30). Eighty-five patients (78%) received care of uncertain concordance during 1 or more epochs, although it was significantly less frequent among patients who received a palliative care consultation during the encounter (26 patients [60%] vs 59 patients [89%]; *P* < .001; χ^2^ = 12.69) and among patients with a diagnosis of metastatic cancer (34 patients [68%] vs 51 patients [86%]; *P* = .02; χ^2^ = 5.36). Finally, among the 83 patients (76%) with 1 or more epochs during the study period (ie, ≥1 GOC discussion documented), 76 (92%) experienced variable care concordance over time (Figure 3). For example, 43 of 68 patients (63%) whose initial epoch was classified as uncertain concordance subsequently received either GCC (27 patients [63%]) or goal-discordant care (16 patients [37%]).

## Discussion

We conducted a retrospective cohort study to demonstrate feasibility of implementing a longitudinal GCC measurement approach using electronic clinical notes and documented GOC discussions as guideposts for epochs of care. We found that clinician reviewers could confidently classify the overall intent of care for more than 95% of epochs with high interrater reliability, thus enabling determination of whether the care patients received was concordant with their documented GOC. The reliability of this approach is further supported by the findings of positive associations between a high rate of GCC and several clinical characteristics associated with a greater frequency of documented GOC discussions, such as palliative care consultation, higher mortality risk, and metastatic cancer. These results suggest that an EHR-based measurement approach has great promise to improve the pragmatism and reliability of the elusive, yet highly valued, serious illness outcome of GCC compared with other approaches that rely on patient, caregiver, or clinician reports.^22^ This proof-of-principle study provides findings that address 4 key knowledge gaps in GCC outcomes research.

First, nearly three-fourths of patients received GCC and more than one-third received goal-discordant care for at least 1 period during their care over 6 months. This demonstrates that the concordance, or lack thereof, between patients’ goals and the care they receive is often dynamic, suggesting that a cross-sectional approach to measuring GCC is suboptimal and risks outcome misclassification.^12,16^ Prior longitudinal assessments of GCC have been limited by temporal gaps between goals and treatment assessment,^11^ which ignores the fact that patients’ goals can change over time.^23^ Our novel approach overcame this limitation by using consecutively documented GOC discussions as they occurred naturally over time, and the 1-week median duration of an epoch of care among this seriously ill cohort suggests that most GCC assessments were based on patients’ current GOC.

Second, the same treatment for patients with different GOC may be goal concordant in both cases. For example, we found cases in which paracenteses performed to palliate abdominal pain aligned with some patients’ comfort-focused goals and others that were performed to diagnose the cause of shock aligned with goals of life extension or maintain or improve function. Prior GCC studies that used existing data assessed alignment of specific treatments with patients’ goals without considering the clinical context and intent with which they were provided.^11^ Our findings suggest that this narrow approach risks outcome misclassification, and, for this reason, it is important to assess the care received through comprehensive review of all available clinical note data. Machine learning and natural language processing methods are well-suited to efficiently accomplish this task.

Third, goal-discordant care was identified more commonly when patients’ GOC were to maintain and improve function. The less circumscribed nature of a functional goal compared with a comfort-focused or life-extension goal may have made it more challenging for reviewers to determine whether the care received was aligned. We also showed in prior work^18^ that patients may concurrently have goals of improving function and living longer, suggesting that care received may not need to align exclusively with 1 GOC to indicate GCC. Indeed, nearly all care that is consistent with a goal to maintain and improve function may also be consistent with the goal of life extension, because after all, one must be alive to function. However, there is likely a point at which the specific treatment being considered primarily serves 1 goal at the expense of another (eg, resuscitation in the event of cardiac arrest). More work is needed to further define common function-focused goals and to expand this method to account for overlapping GOC when measuring GCC that acknowledges the nuances of serious illness care.

Fourth, we identified several hypothesis-generating clinical findings. Patients who received a palliative care consultation were more likely to receive GCC for much of the follow-up period and were less likely to receive care of uncertain concordance. These findings strengthen the construct validity of our measurement approach and align with prior studies showing that palliative care consultation leads to increased documentation of GOC discussions that is presumably of high quality, thus enabling greater opportunity to provide (and measure) GCC.^20,24,25^ We also found that patients admitted to a medical service had higher rates of goal-discordant care compared with those admitted to a surgical service. One possible explanation is that admissions to a surgical service are more likely to involve a planned procedure that was determined in advance to be aligned with patients’ goals. Alternatively, goal-discordant care may have been underrecognized in surgical patients in this cohort owing to their lower rates of GOC documentation. Finally, the finding that patients with metastatic cancer had higher rates of both GCC and goal-discordant care over time warrants further investigation.

This study has several strengths. First, we broadly defined serious illness to promote generalizability of our approach and findings among a typical hospitalized population. Second, our novel longitudinal approach using clinical epochs defined by GOC discussions reflects the real-world shifts in an individual’s experience of serious illness care over time. Third, we used blinded, dual-record review to mitigate reviewer bias and promote veracity of our findings. Finally, demonstrating feasibility of this novel EHR-based approach to identify GCC serves as important proof of concept for the promise of natural language processing and machine learning methods to augment widespread applicability.^26,27,28^

### Limitations

These findings should be interpreted considering several limitations. First, we conducted this work in a single health system. Local culture can influence clinicians’ GOC communication and documentation as well as serious illness treatment practices. External validation of this approach in another health system is needed. Second, we relied on reviewers’ interpretation of the primary intent of care received from EHR documentation alone. Misclassification of care received because of incomplete documentation is possible. A prospective validation study would enable a comparison of the reviewers’ classification of care received with the true intent of care reported by treatment team. Relatedly, future prospective work should include validation from the patient or family of the classification of GCC from EHR data. Third, it is important to acknowledge the trade-off of individualization when categorizing GOC and care received to facilitate pragmatic measurement of GCC. Distillation of individuals’ unique and sometimes complex goals (eg, multiple goals concurrently or contingent goals) into few discrete categories may overlook the personalization of the care they received, leading to misclassification of goal-discordant care.^3,16,17,18,22^ Next steps in GCC classification models may need to allow for greater flexibility in assigning care concordance for multiple concurrent or contingent GOC (eg, time-limited trials). Fourth, manual record review was necessary for this proof-of-concept study, but it is highly resource intensive. Future efforts in larger cohorts ought to use widely available natural language processing classification methods for practicability,^26,29,30,31^ which could also enable a more granular assessment of GCC by day rather than by epoch.

## Conclusions

In this cohort study of seriously ill adults with limited prognoses, we demonstrated that the type of care they received can be classified into distinct categories, thereby enabling the measurement of GCC using EHR clinical note data. The validity of this novel approach was further supported by findings of positive associations between receipt of GCC and several clinical characteristics associated with the timing and quality of serious illness communication.^9^ As automated text-based classification methods are increasingly integrated into EHRs and used to identify GOC,^26,27,28,29,31,32,33^ the findings from this novel approach to measuring GCC may be applied to support widespread clinical and research use.

## Notes

Supplement 1. **eTable 1.** Cohort characteristics **eTable 2.** Distribution of epochs by categories of care received and goals of care

Supplement 2.
Data Sharing Statement
