# Supplementary material for: Measuring Goal-Concordant Care Using Electronic Clinical Notes
Source: JAMA Netw Open. 2025 Jul 3;8(7):e2518967. doi: 10.1001/jamanetworkopen.2025.18967 (PMC12232218; doi:10.1001/jamanetworkopen.2025.18967)
Supplement: Supplement 1. — eTable 1. Cohort characteristics eTable 2. Distribution of epochs by categories of care received and goals of care [file jamanetwopen-e2518967-s001.pdf]

Supplemental Online Content

Auriemma CL, Song A, Walsh L, et al. Measuring goal-concordant care using electronic clinical notes. *JAMA Netw Open*. 2025;8(7):e2518967. doi:10.1001/jamanetworkopen.2025.18967

eTable 1. Cohort characteristics

eTable 2. Distribution of epochs by categories of care received and goals of care

This supplemental material has been provided by the authors to give readers additional information about their work.

© 2025 Auriemma CL et al. *JAMA Network Open*.

eTable 1. Cohort characteristics

Clinical Variable [a]	All patients (n = 109)
Age, years	70 (63, 79)
Female sex, %	53 (49)
Race	
White, %	63 (58)
Black, %	41 (38)
Multi-racial, %	2 (2)
Asian, %	1 (1)
Other, %	2 (2)
Ethnicity	
Not Hispanic/Latinx, %	108 (98)
Prefer not to answer, %	1 (2)
Religion, %	
Christian	40 (37)
Roman Catholic	31 (28)
Jewish	13 (12)
Other	5 (5)
Unknown	20 (18)
Primary language	
English	106 (97)
Other	3 (3)
Insurance	
Medicare	83 (76)
Private	22 (20)
Medicaid	4 (4)
Chronic illness category [b], %	
Cardiac, %	76 (70)
Metastatic cancer, %	50 (45)
Renal, %	42 (39)
Pulmonary, %	23 (21)
Neurologic, %	22 (20)
Liver, %	16 (15)
Lymphoma, %	13 (12)
Solid tumor without metastases, %	10 (9)
Elixhauser score	6 (4, 8)
Predicted six-month mortality	
High 0.5-0.74	54 (50)
Very high 0.75-1	55 (50)
Admit type	
Medical	90 (83)
Surgical	19 (17)
Admission source	
Emergency department	85 (78)
Direct from clinic or home	7 (6)
Another institution	17 (16)
Index hospitalization LOS	7.8 (4.8, 12.0)
Palliative care consultation during index hospitalization	43 (39)
ICU stay during index hospitalization	35 (32)
Palliative care consultation during follow-up	14 (15)
Rehospitalization during follow-up	54 (58)
Home care enrollment during follow-up	61 (66)
Goals of care categorizations (n=338)	
Life prolongation	93 (28%)
Maintain/improve function	110 (33%)
Comfort focused	83 (25%)
Unclear	52 (15%)

[a] Data shown as mean ± standard deviation, number (percent), or median (interquartile range) as appropriate
[b]Disease categories determined based on ICD-10 codes for Elixhauser comorbidity scores

© 2025 Auriemma CL et al. *JAMA Network Open*.

eTable 2. Distribution of epochs by categories of care received and goals of care.

		Care Received				Total
		Life extension	Maintain or improve function	Comfort-focused	Unclear	
Goals of Care	Life extension	71	17	1	4	93
	Maintain or improve function	29	66	9	6	110
	Comfort-focused	3	15	61	4	83
	Unclear	27	23	0	2	52
	Undocumented	53	7	0	0	60
Total		183	128	71	16	398

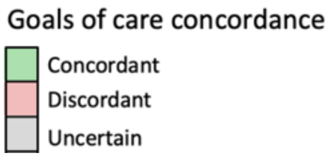

© 2025 Auriemma CL et al. *JAMA Network Open*.
