# Supplementary material for: Measuring Goal-Concordant Care Using Electronic Clinical Notes
Source: JAMA Netw Open. 2025 Jul 3;8(7):e2518967. doi: 10.1001/jamanetworkopen.2025.18967 (PMC12232218; doi:10.1001/jamanetworkopen.2025.18967)
Supplement: Supplement 2. — Data Sharing Statement [file jamanetwopen-e2518967-s002.pdf]

Data Sharing Statement

Auriemma. Measuring Goal-Concordant Care Using Electronic Clinical Notes. *JAMA Netw Open*. Published July 03, 2025. doi:10.1001/jamanetworkopen.2025.18967

Data
Data available: Yes
Data types: Deidentified participant data, Data dictionary
How to access data: Email request to catherine.auriemma@pennmedicine.upenn.edu
When available: With publication

Supporting Documents
Document types: Statistical/analytic code
How to access documents: Email request to catherine.auriemma@pennmedicine.upenn.edu
When available: With publication

Additional Information
Who can access the data: Researchers whose proposed use of the data has been approved.
Types of analyses: For specified research purposes in an approved proposal.
Mechanisms of data availability: After approval of a proposal
